# Geo-Location Information Aided Spectrum Sensing in Cellular Cognitive Radio Networks †

**DOI:** 10.3390/s20010213

**Published:** 2019-12-30

**Authors:** Siji Chen, Bin Shen, Xin Wang, Sang-Jo Yoo

**Affiliations:** 1 School of Communication and Information Engineering (SCIE), Chongqing University of Posts and Telecommunications (CQUPT), Chongqing 400065, China; cqchensj@outlook.com (S.C.); s170101160@stu.cqupt.edu.cn (X.W.); 2 Department of Information and Communication Engineering, Inha University, Incheon 402751, Korea; sjyoo@inha.ac.kr

**Keywords:** spectrum sensing, geolocation, wireless fingerprint database, support vector machine, dynamic spectrum access, cognitive radio

## Abstract

Apart from the received signal energy, auxiliary information plays an important role in remarkably ameliorating conventional spectrum sensing. In this paper, a novel spectrum sensing scheme aided by geolocation information is proposed. In the cellular cognitive radio network (CCRN), secondary user equipments (SUEs) first acquire their wireless fingerprints via either received signal strength (RSS) or time of arrival (TOA) estimation over the reference signals received from their surrounding base-stations (BSs) and then pinpoint their geographical locations through a wireless fingerprint (WFP) matching process in the wireless fingerprint database (WFPD). Driven by the WFPD, the SUEs can easily ascertain for themselves the white licensed frequency band (LFB) for opportunistic access. In view of the fact that the locations of the primary user (PU) transmitters in the CCRN are either readily known or practically unavailable, the SUEs can either search the WFPD directly or rely on the support vector machine (SVM) algorithm to determine the availability of the LFB. Additionally, in order to alleviate the deficiency of single SUE-based sensing, a joint prediction mechanism is proposed on the basis of cooperation of multiple SUEs that are geographically nearby. Simulations verify that the proposed scheme achieves higher detection probability and demands less energy consumption than the conventional spectrum sensing algorithms.

## 1. Introduction

With the aim of identifying the status of the licensed spectrum and enabling secondary access, spectrum sensing techniques have been extensively investigated for cognitive radio (CR) in recent years [1,2,3,4,5]. In order to guarantee that, as little interference is generated to the primary user (PU) as possible, secondary users (SUs) in the cognitive radio network (CRN) can only gain opportunistic access to the licensed frequency band (LFB) when they detect no PU activity over it. However, the task of ascertaining PU behavior over the licensed spectrum is practically challenging if conventional spectrum sensing methods, e.g., energy detection (ED), are concerned, since the signals transmitted from the PUs are usually subject to complicated radio propagation effects before reaching the SUs, especially when the PU signals encounter severe power attenuation and fast fluctuations. In addition, if it is assumed that the SUs in the CRN are cellular secondary user equipments (SUEs) (Correspondingly, the CRN is referred to as the cellular cognitive radio network (CCRN) hereinafter.) periodically collecting spectrum observations over the LFB and reporting them to their serving base-stations (BSs) for global decisions on the status of the LFB, it would probably arouse another problematic issue of power deficiency for battery driven SUE devices. In particular, the battery time that the SUEs could sustain may be significantly shortened due to the dual-band power consumption.

In the existing literature, the spectrum sensing techniques can be roughly categorized into four types. Firstly, threshold-test-based signal detection, such as ED [6,7,8], cyclostationary feature detection [9,10,11], and matched filtering detection [12,13,14] serve as common methods for the SUEs to gain awareness of the LFB’s PU occupancy status. Secondly, for achieving higher accuracy and reliability, cooperative spectrum sensing (CSS) algorithms have been extensively investigated [15,16,17,18], where space diversity in the CRN is exploited and different hard decision fusion (HDF) schemes and soft data fusion algorithms are proposed under different criteria [15]. Thirdly, different from the single/multi-user-based signal detection schemes, some hybrid spectrum sensing strategies that combine signal processing techniques and geolocation databases have been proposed [19,20,21]. The geolocation database stores in advance some information about the PUs, such as the PU transmitter’s (PUT’s) power, geographical position, statistical behavior, and so forth. Supported by this additional a priori information, the database-based sensing approaches substantially relieve the complexity requirement and power demand on the SUEs’ devices [22,23]. Fourthly, as one kind of promising solution, machine learning-based spectrum sensing (MLSS) schemes have also been researched in recent years [24,25,26,27,28], where it has been testified that some typical supervised and unsupervised machine learning methods work well in discerning the spectrum observations collected from different situations and even outperform some conventional CSS methods, for instance, the AND, OR, and Majority criteria-based HDF schemes. It is of special importance to note that the conventional threshold-test methods, CSS schemes, and MLSS algorithms usually operate on the spectrum observations only and aim to detect the weakest PU signal possible. It certainly helps significantly in relieving the hidden terminal effect, but the spectrum opportunity is unfortunately ignored when the SUEs are sufficiently far away from the PUs. On the other hand, the conventional geolocation database supported sensing method operates on the statistical knowledge that is drawn over a relatively long period. It, therefore, lacks the flexibility of being able to adapt to the transient behavior of the PUs and it may even fail to achieve the sensing agility requirement if a plug-and-play sensing method is demanded.

In order to strengthen the SUEs’ capability of squeezing the opportunistic spectrum chances and enhance their energy efficiency in sensing, we propose a geolocation information aided spectrum sensing scheme on the basis of the wireless fingerprint database (WFPD) and machine learning (ML) algorithms. The philosophy of resorting to WFPD and ML algorithms in the proposed sensing scheme is twofold. Firstly, the WFPD helps the SUEs a lot in easily identifying their own geographical locations and hence they are able to search the database and easily obtain the LFB availability information corresponding to their current positions in the CCRN. This mechanism is of great benefit for the SUEs’ energy efficiency in sensing because the conventional periodic acquisition of spectrum observations over the LFB is circumvented in a proactive manner. In this way, a large portion of energy consumed in scanning the LFB can be saved. Secondly, assisted by the information and data stored in the WFPD, it is easier for the SUEs to take spectrum sensing decisions when ML methods are adopted for processing the spectrum observations with high efficiency and precision. Furthermore, and to be more specific, aided by the WFPD, geolocation related spectrum availability for a specific SUE can be achieved according to the distance between the SUE and the PUTs. This geolocation information enables the SUEs to suppress the nuisance interference generated by themselves to the PU receivers in their vicinity. In practice, however, locations of the PUTs can be either readily known or completely unknown [29]. For the two different situations, wireless fingerprint (WFP) searching-based sensing scheme and the MLSS algorithm-based HDF scheme are proposed. It is verified in simulations that the proposed scheme outperforms the conventional HDF-based CSS algorithms in terms of sensing performance. Meanwhile, the proposed scheme also achieves higher energy efficiency than the conventional periodic spectrum sensing schemes.

The rest of this paper is organized as follows. In Section 2, we describe the CCRN and present the signal model. Section 3 briefly reviews the non-geolocation information aided spectrum sensing methods. In Section 4, we propose the geolocation information aided spectrum sensing schemes for different scenarios. In Section 5, performance evaluation results for the proposed schemes are presented. Finally, Section 6 concludes the paper.

## 2. System Model

### 2.1. Cellular Cognitive Radio Network

We consider a CCRN, where there are multiple BSs serving a number of SUEs over the CCRN’s own frequency band denoted by $F_{1}$. By means of any possible strategy or mechanism, the SUEs attempt to seize possible opportunities of accessing the LFB, denoted as $F_{2}$, to offload the traffic that could not be undertaken over their own frequency band $F_{1}$.

The target geographic area of the CCRN is divided into *Q* grids with the same area (The area of the grids reflects the spatial resolution requirement in status identification of the LFB.), where any specific SUE in the CCRN can be located in terms of the grid it is currently camping in. If one database is established in advance, with its data tables storing information about the availability of the LFB within each specific grid in the CCRN, then the problem of spectrum sensing could be easily solved by first positioning the SUEs and then searching the database for the pre-stored spectrum availability information. This kind of database-driven spectrum sensing mechanism is of particular benefit in practice. Normally as battery supported devices communicating with the BSs over $F_{1}$ and simultaneously monitoring $F_{2}$, the SUEs in the CCRN inevitably encounter a problem of power deficiency, which is a critical issue to be tackled. In this sense, with the support of the database, the SUEs are provided an option of determining the status of $F_{2}$ via simple database operations.

### 2.2. Signal Model

It is assumed that *K* BSs continuously operate over $F_{1}$ in the CCRN and simultaneously *P* PUTs serve their PU receivers over $F_{2}$ within the same geographic area. When communicating with its serving BS (the *k*-th BS) over $F_{1}$, the SUE in the *q*-th grid (Without loss of generality, we refer to the SUE in the *q*-th grid as the *q*-th (q=1,2,⋯,Q) SUE in this paper, even though in practice there may be multiple SUEs camping in the *q*-th grid  simultaneously.) receives the signal as

(1) $${\bar{y}}_{qk}\left(i\right)=\sqrt{{\bar{E}}_{k}}{\bar{h}}_{kq}{\bar{s}}_{k}\left(i\right)+\sum_{j=1,j\neq k}^{K}\sqrt{{\bar{E}}_{j}}{\bar{h}}_{jq}{\bar{s}}_{j}\left(i\right)+{\bar{n}}_{q}\left(i\right),$$

where *i* is the time index for signal samples, ${\bar{E}}_{k}$ is the transmit power of the *k*-th BS, ${\bar{h}}_{kq}$ is the channel coefficient from the *k*-th BS to the *q*-th SUE, ${\bar{s}}_{k}$ is the downlink signal transmitted by the *k*-th BS, and ${\bar{n}}_{q}\left(i\right)∼\mathrm{CN}(0,{\bar{\sigma }}_{0}^{2})$ is the complex additive white Gaussian noise (AWGN) corresponding to the *q*-th SUE in the $F_{1}$ band.

In addition to communicating over $F_{1}$, the *q*-th SUE keeps repeatedly observing the LFB $F_{2}$ with the sensing period $T_{s}$ in conventional sensing methods. It obtains the signal received from the PUTs in the *m*-th sensing operation as

(2) $$y_{qm}\left(i\right)=\left\{\begin{array}{c} \sum_{p=1}^{P}\sqrt{I^{+}\left(E_{p}^{\left(m\right)}\right)}h_{pq}^{\left(m\right)}x_{p}^{\left(m\right)}\left(i\right)+n_{q}^{\left(m\right)}\left(i\right),\;H_{1}\\ n_{q}^{\left(m\right)}\left(i\right),\;\;\;\;\;\;\;\;\;\;\;\;\;\;\;\;\;\;\;\;\;\;\;\;\;\;\;\;\;\;\;\;\;\;\;\;\;\;\;\;\;\;\;\;\;\;\;H_{0} \end{array}\right.$$

where *i* stands for the time index within the *m*-th spectrum observation, $I^{+}\left(E_{p}^{\left(m\right)}\right)=\max\left(E_{p}^{\left(m\right)},0\right)$ with $E_{p}^{\left(m\right)}\in \left\{E_{p},0\right\}$ referring to the transmit power level (For the purpose of simplicity, we assume in this paper that the PUTs have only two possible transmit power levels, although, in practice, there are usually multiple transmit power levels that need to be identified.) of the *p*-th PUT, $h_{pq}^{\left(m\right)}$ denotes the channel coefficient from the *p*-th PUT to the *q*-th SUE, $x_{p}^{\left(m\right)}\left(i\right)$ is the transmit symbol of the *p*-th PUT, $n_{q}^{\left(m\right)}\left(i\right)∼\mathrm{CN}(0,\sigma_{0}^{2})$ is the complex AWGN of the *q*-th SUE in the $F_{2}$ band, and the hypotheses $H_{1}$ and $H_{0}$, respectively, represent the case of at least one PUT being busy and the case that all the PUTs are idle.

The channel coefficient $h_{pq}^{\left(m\right)}$ can be modeled as

(3) $$h_{pq}^{\left(m\right)}=\sqrt{\mathrm{PL}(∥C_{p}^{\mathrm{PUT}}-C_{q}^{\mathrm{SUE}}∥)\;}\;\cdot \zeta_{p}\cdot \nu_{p},$$

where $C_{p}^{\mathrm{PUT}}={\left[C_{p,x}^{\mathrm{PUT}}\;C_{p,y}^{\mathrm{PUT}}\right]}^{T}$ stands for the position of the *p*-th PUT, $C_{q}^{\mathrm{SUE}}={\left[C_{q,x}^{\mathrm{SUE}}\;C_{q,y}^{\mathrm{SUE}}\right]}^{T}$ is the position of the *q*-th SUE, ∥.∥ is the Euclidean distance, $\mathrm{PL}\left(d\right)=d^{-a}$ is the path-loss component for the relative distance *d* with the path-loss exponent *a*, $\zeta_{p}$ is the shadowing component, and $\nu_{p}$ is the multipath fading component in accordance with Rayleigh distribution.

Within the *m*-th sensing operation, the *q*-th SUE acquires the spectrum observation vector as $y_{qm}\;=\;{\left[y_{qm}\left(1\right),y_{qm}\left(2\right),\cdots \;,y_{qm}\left(2W\tau \right)\right]}^{T}$, where *W* is the PU signal bandwidth in $F_{2}$ and τ is the sensing interval. Over *M* consecutive sensing interval τ within each $T_{s}$, the aggregate observations can be organized as $Y_{q}=\left[y_{q1},y_{q2},\cdots ,y_{q\;M}\right]$, where *M* is the total number of spectrum observation segments. Based on the raw data $Y_{q}$, the spectrum occupancy status of $F_{2}$ with respect to the *q*-th grid can be decided, through either conventional signal detection methods or MLSS algorithms. It is noteworthy that depending on the grid granularity in the CCRN, some neighboring grids may obtain the same spectrum decisions. Once all the grids requesting for spectrum opportunities are aware of their own spectrum status of $F_{2}$, the spectrum sensing task is fully fulfilled in the CCRN.

## 3. Non-Geo-Location Information Aided Spectrum Sensing

### 3.1. Conventional Threshold-Test-Based Spectrum Sensing Methods

#### 3.1.1. Single User-Based Energy Detection

According to the well-known ED, the *q*-th SUE measures the received signal energy in $y_{qm}$ as

(4) $$Y_{qm}={∥y_{qm}∥}^{2}=\sum_{i=1}^{2W\tau }{\left|y_{qm}\left(i\right)\right|}^{2},$$

and obtains the test statistic $Z_{q}$ and the spectrum decision $D_{q}$ via the threshold-test

(5) $$\begin{array}{c} Z_{q}=tr\left(Y_{q}Y_{q}^{H}\right)=\sum_{m=1}^{M}{∥y_{qm}∥}^{2}≷\lambda_{q}\begin{array}{cc} ↗\; & D_{q}={\hat{H}}_{1},\\ ↘\; & D_{q}={\hat{H}}_{0}, \end{array} \end{array}$$

where $\lambda_{q}$ is the pre-calibrated threshold, depending on the desired false alarm probability under the criterion of constant false alarm probability (CFAP), $∥\;.\;∥$ is the vector norm, tr(.) is the matrix trace operator, and the test decision $D_{q}$ is either ${\hat{H}}_{0}=0$ for the case of all PUTs being idle or ${\hat{H}}_{1}=1$ for the case of any PUT being active in the CCRN, respectively.

#### 3.1.2. Multi-User-Based HDF Sensing

On obtaining the spectrum decision $D_{q}$, the *q*-th SUE may cooperate with multiple SUEs in its neighbourhood to strengthen the sensing reliability through the HDF criteria, e.g., AND, OR, and Vote, as

(6) $$\begin{array}{c} \Lambda_{q}\;=\sum_{t\in Q_{q},t\neq q}D_{t}+D_{q}≷\eta \begin{array}{cc} ↗\; & {\tilde{D}}_{q}={\hat{H}}_{1},\\ ↘\; & {\tilde{D}}_{q}={\hat{H}}_{0}, \end{array} \end{array}$$

where $D_{t}$ is the spectrum decision of the *t*-th SUE, $Q_{q}$ is the index set of the *q*-th SUE’s neighboring SUEs with the set cardinality denoted as $|Q_{q}|$, and η is an integer threshold for the HDF schemes. It can be observed that the HDF scheme boils down to the AND scheme for $\eta =|Q_{q}|+1$, the OR scheme for η=1, and the $\eta_{0}$-out-of-$\left(|Q_{q}|+1\right)$ (a.k.a Vote) scheme for $\eta =\eta_{0}$, respectively.

The sensing performance is usually quantified in terms of receiver operating characteristics (ROC), which gives the detection probability $P_{D}=Pr\left({\hat{H}}_{1}\;|\;H_{1}\right)$ as a function of the probability of false alarm $P_{\mathrm{FA}}=Pr\left({\hat{H}}_{1}\;|\;H_{0}\right)$. By varying the detection threshold, the operating point of a detector can be chosen anywhere along its ROC curve.

### 3.2. Machine Learning-Based Spectrum Sensing

In recent years, machine learning-based classification algorithms have been proposed to identify the LFB status for CR systems, since the ultimate task of sensing is in some sense equivalent to classifying the spectrum observations as the data containing PU signal components or the data composed of noise only. Typically, ML algorithm such as support vector machine (SVM) and clustering algorithms can be utilized for spectrum sensing. For the purpose of simplicity, we assume in this paper that the transmit powers of the PUTs are fixed within each sensing period $T_{s}$, i.e., $E_{p}^{\left(m\right)}=E_{p,h}$ for all m=1,2,⋯,M, and the *p*-th PUT has only two possible power levels $E_{p,h}\in \left\{E_{p},0\right\}$. Practically in the *m*-th sensing operation, the *P* PUTs may operate in mutually independent modes and hence there are in total $2^{P}$ operation states of the PUTs in the CCRN, where for the *h*-th state, the corresponding power vector of the PUTs can be expressed as

(7) $$P_{h}={\left[I^{+}\left(E_{1,h}\right),I^{+}\left(E_{2,h}\right),\cdots ,I^{+}\left(E_{p,h}\right),\cdots ,I^{+}\left(E_{P,h}\right)\right]}^{T},\;h\in \left\{0,1,\cdots ,2^{P}\;-\;1\right\}.$$

For the SVM-based sensing data classifier, we can get the *q*-th energy vector from Equation (4) within each sensing period $T_{s}$ as

(8) $$\theta_{q}={\left[\;Y_{q1},Y_{q2},\cdots ,Y_{qM}\;\right]}^{T},$$

where $Y_{qm}$ is the received signal energy captured for the *q*-th SUE.

Prior to feeding $\theta_{q}$ into the SVM, we need to obtain enough number of energy vectors to form the training data set. Given a sufficiently large number of energy vectors that may be accumulated in a relatively long time, we utilize the training data set and the training label set as

(9) $$\begin{array}{cc} \Theta_{q,L} & =\left\{\theta_{q}^{\left(1\right)},\theta_{q}^{\left(2\right)},\cdots ,\theta_{q}^{\left(l\right)},\cdots ,\theta_{q}^{\left(L\right)}\right\},\\ C_{q,L} & =\left\{C_{q}^{\left(1\right)},C_{q}^{\left(2\right)},\cdots ,C_{q}^{\left(l\right)},\cdots ,C_{q}^{\left(L\right)}\right\}, \end{array}$$

where *L* is the number of energy vectors adopted for training and the corresponding label $C_{q}^{\left(l\right)}$ is either ${\hat{H}}_{0}$ or ${\hat{H}}_{1}$. After the SVM classifier is successfully trained, it can be used to classify the spectrum observations. In addition, it is worth noting that for conventional ED and MLSS schemes, only when the PUTs are operating in the h=0 state, i.e., $P_{0}={\left[0,0,\cdots ,0\right]}^{T}$, the label $C_{q}^{\left(l\right)}$ can be set as ${\hat{H}}_{0}$. It is apparent that in fact the state $P_{0}={\left[0,0,\cdots ,0\right]}^{T}$ means that no active PUT is found in the CCRN at all and it is the only state under which the SUEs are allowed to access the LFB $F_{2}$. Therefore, it is actually an over-strict condition for the SUEs to gain spectrum opportunities over $F_{2}$ only in case that the PUTs’ state is identified as $P_{0}$, whereas the spectrum opportunities possibly hidden in the states $P_{h},h\in \left\{1,2,\cdots ,2^{P}-1\right\},$ are ignored.

In the MLSS schemes, when the test data $\theta_{q}$ is fed into the readily trained classifier and the label allocated to it is ${\hat{C}}_{q}$, the spectrum sensing accuracy can be evaluated as

(10) $$P_{A}=\sum_{p=1}^{2}E_{q}\left[Pr({\hat{C}}_{q}=C_{q}|H_{p})Pr\left(H_{p}\right)\right]\approx \sum_{p=1}^{2}\frac{1}{Q}\sum_{q=1}^{Q}I({\hat{C}}_{q}=C_{q})Pr\left(H_{p}\right),$$

where $H_{p}$ has only two possible hypothesis as $H_{1}$ and $H_{2}$, $C_{q}$ is the true label of $\theta_{q}$, E[.] is the mathematical expectation, Pr(A) is the probability of an event *A*, and I(.) is the indicator function.

## 4. Geo-Location Information Aided Spectrum Sensing

In the previous section, the conventional signal detection-based sensing schemes and the MLSS algorithms obtain the spectrum decisions from the spectrum observations only. Without additional information that may be helpful in sensing, the aforementioned schemes can merely acquire the spectrum decisions under the very stringent constraint of ascertaining whether PU signal components exist in the spectrum data under test. Drawbacks of these methods are apparent. Firstly, a relatively large portion of the potential spectrum opportunities in the CCRN may be wasted. Even when the SUE is sufficiently far away from the active PUTs, it can not be granted permission to access $F_{2}$. Interestingly, this problem may occur when cooperation of some SUEs is evoked to enhance the reliability in sniffing the weak PU signals (Bearing in mind that hidden terminal effect may be harmful, we usually find that the conventional spectrum sensing schemes are designed to prevent the SUEs from accessing the LFB when weak PU signals are detected in the spectrum observations.). Secondly, without offline history information of the spectrum status, the SUEs need to repeatedly monitor the LFB $F_{2}$, even when its status is relatively stable or only varying slowly over time. This case actually imposes a heavy burden on the power consumption of the SUEs.

Aiming to tackle these problems encountered in conventional spectrum sensing schemes, we propose to exploit geolocation information to assist the SUEs in sensing operations. Tailored for geolocation information-based sensing scheme, the WFPD can be viewed as an indispensable infrastructure consisting of a large variety of different data. Specifically, aided by the WFPD, the SUEs are easy to locate themselves in the CCRN, with short time and high precision. When the positions of the PUTs are possibly available to the SUEs and the WFPD stores records of the PU behaviors, the SUEs are capable of quickly identifying the LFB availability with respect to their own locations.

### 4.1. Geographical Region-Based Spectrum Status Identification

**Figure 1:**
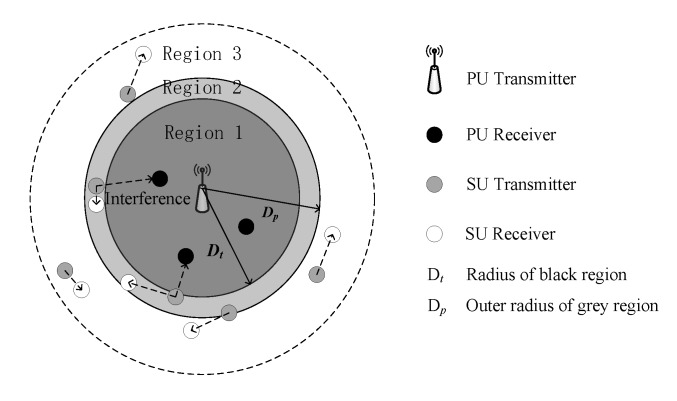
Geo-regions-based spectrum status identification.

According to the robustness requirement of spectrum sensing stipulated in the IEEE 802.22 standard [30], a region division strategy over the active PUTs is considered in the CCRN. The target geographical area is divided into black, grey, and white regions, as shown in Figure 1 for example. Centered with an active PUT, the black region with radius $D_{t}$ is closely surrounded by the grey region with inner radius $D_{t}$ and outer radius $D_{p}$. Meanwhile, the grey region is surrounded by the white region. Inside the black region, the PUTs have exclusive right to use the LFB $F_{2}$ and the SUEs are absolutely forbidden to operate over $F_{2}$, thus tremendously eliminating any possible interference to the PU receivers inside it. The temporal spectrum opportunities may be found in the grey region, where the SUEs can opportunistically gain access, and in the white region, where the SUEs are of sufficiently long distances from the PU receivers and therefore are allowed to transmit with their maximum power at any time, without causing severe interference to the PU receivers.

**Figure 2:**
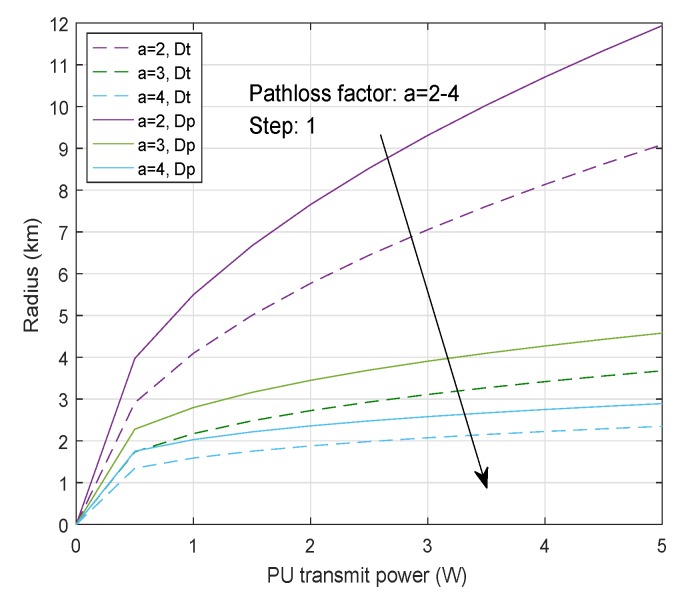
Relationship between the region radius and primary user transmitter (PUT) power.

Specifically, radius of the black region surrounding the *p*-th active PUT can be defined as [31]

(11) $$D_{t}\leq \beta_{t}{\left(\frac{E_{p}}{\sigma_{0}^{2}\left(2^{\xi_{0}}-1\right)}\right)}^{1/a}$$

where $\xi_{0}$ is a threshold constant and $\beta_{t}$ is an adjusting coefficient. The outer radius of the gray region is

(12) $$D_{p}={\left[\frac{2E_{p}WM\tau }{\sigma_{0}^{2}\left(q_{f}\sqrt{2WM\tau }+q_{m}^{2}+q_{m}\sqrt{2WM\tau +2q_{f}\sqrt{2WM\tau }+q_{m}^{2}\;}\;\;\right)}\right]}^{1/a}$$

where $q_{f}=Q^{-1}\left(\varepsilon_{f}\right)$, $q_{m}=Q^{-1}\left(\varepsilon_{m}\right)$, the parameter $\varepsilon_{f}$ and $\varepsilon_{m}$ are constraint constants of $P_{\mathrm{FA}}$ and $P_{M}$ (probability of miss detection), respectively, with $P_{\mathrm{FA}}\leq \varepsilon_{f}$, $P_{M}\leq \varepsilon_{m}$ required, and $Q^{-1}\left(.\right)$ is the inverse *Q*-function. For the purpose of ensuring the quality of PU communication and guaranteeing the spectrum opportunities for the SUEs, the region partitioning can be flexibly adjusted, as shown in Figure 2.

The spectrum availability of $F_{2}$ in different regions can be denoted by the flags given in Table 1, where the PUT_Flag is set identical with the Region_Flag. The PUT_Flag with value 1 means no spectrum opportunity over $F_{2}$ is found for the SUEs, -1 means the SUEs are safe to transmit over $F_{2}$, and 0 implies potential spectrum opportunities might be discovered in the grey region. As an enabling label for the SUEs to access $F_{2}$, the next step of spectrum sensing is to identify the region that the SUEs are camping in.

### 4.2. WFPD Aided SUE Positioning

After defining the regions in the CCRN, the next step of spectrum sensing is to find the locations of the SUEs. When the SUEs are able to know their own locations, they can make a quick spectrum decision based on the spectrum availability information stored in the WFPD. Since it is well known that wireless positioning technology based on received signal strength (RSS) and time of arrival (TOA) are common methods that may be utilized practically, they are both employed in this paper. The TOA positioning technique refers to the method of estimating the time that the BS downlink reference signal takes to arrive at the SUE. Observing the surrounding BSs’ downlink reference signals in routine operations such as synchronization tracking and reference signal received power (RSRP) measurement, the SUEs can measure the downlink reference signals’ RSS and TOA of neighboring BSs to locate themselves in the CCRN [32,33].

Based on the received signal in Equation (1), the *q*-th SUE utilizes the locally generated reference signal over $F_{1}$ to seek the peak of the correlation output as the TOA. The signal correlation is first obtained as

(13) $$R_{qk}\left(l\right)={\left|\sum_{l_{1}=1}^{L_{1}}{\bar{y}}_{qk}\left(l+l_{1}\right)\cdot r_{q,\mathrm{local}}\left(l_{1}\right)\right|}^{2},$$

where ${\bar{y}}_{qk}={\left[{\bar{y}}_{q1},{\bar{y}}_{q2},\cdots ,{\bar{y}}_{qL_{2}}\right]}^{T}$ is the *k*-th BS’s downlink reference signal received by the *q*-th SUE, ${\bar{y}}_{qk}\left(l+l_{1}\right)$ denotes the $\left(l+l_{1}\right)$-th signal element in ${\bar{y}}_{qk}$, $r_{q,\mathrm{local}}={\left[{\bar{r}}_{q1},{\bar{r}}_{q2},\cdots ,{\bar{r}}_{qL_{1}}\right]}^{T}$ is the local signal generated by the *q*-th SUE, $r_{q,\mathrm{local}}\left(l_{1}\right)$ is the $l_{1}$-th signal sample, and the parameters $L_{1}$ and $L_{2}$ are, respectively, the length of the local signal and the length of the received signal.

Therefore, the estimate of TOA of the *k*-th BS at the *q*-th SUE is obtained as

(14) $$T_{qk}=\frac{1}{F_{s}}\underset{l}{\arg\max}\left(R_{qk}\left(l\right)\right),$$

where $F_{s}$ is the sampling frequency of the BS’s downlink signal.

Before the SUEs can truly perform sensing operations, it is assumed that one WFPD has already been established in advance, as shown in Table 2, and it is fully accessible for all the SUEs in the CCRN. Since there are *Q* grids in the CCRN, the number of WFPs in the WFPD is set as *Q*, too. Each WFP consists of the TOA estimations (For the case of RSS information-based WFPD, RSS data is used instead.) with respect to the *K* BSs, the corresponding *K* BS-IDs, PUT_Position_Flag, Region_Flag, the LFB $F_{2}$’s availability flag (PUT_Flag), the LFB $F_{2}$’s status update timer (Update_Timer), received signal ${\left\{y_{qm}\right\}}_{m=1}^{M}$ and signal energy ${\left\{Y_{qm}\right\}}_{m=1}^{M}$. In addition, there is a public flag PUT_State_Flag_*h* among the *Q* WFPs, where *h* indicates that all the data in Table 2 are actually obtained from the PUTs’ power state $P_{h}$.

If the *q*-th SUE is triggered to sense the LFB $F_{2}$, it first sends its TOA estimations, i.e., the TOA fingerprint, to the WFPD. The TOA fingerprint is a combination of the estimated TOAs and the corresponding BS-IDs, e.g., the $q^{*}$-th WFP ${\left\{{\mathrm{TOA}}_{kq^{*}}\right\}}_{k=1}^{K}$. The *q*-th SUE is located in the $q^{*}$-th grid based on the WFP search when its WFP best matches the $q^{*}$-th WFP in the WFPD as

(15) $$q^{*}\;=\;\;\underset{q^{\prime }\in \left\{1,2,\cdots ,Q\right\})}{\arg\min}\beta_{q^{\prime }}=\;\;\underset{q^{\prime }\in \left\{1,2,\cdots ,Q\right\})}{\arg\min}\sqrt{\;\sum_{k=1}^{K}{\left[T_{qk}-{\mathrm{FP}}_{q^{\prime }}\left(k\right)\right]}^{2}\;},$$

where $\beta_{q}^{\prime }$ is the root square of the TOA estimation error, $q^{*}$ is the index of the WFP that best matches the fingerprint reported by the *q*-th SUE, and ${\mathrm{FP}}_{q^{\prime }}\left(k\right)$ represents the *k*-th fingerprint feature, ${\mathrm{TOA}}_{kq^{\prime }}$ in Table 2, i.e., the TOA estimation of the reference signal from the *k*-th BS to the $q^{\prime }$-th grid in the CCRN. In addition, Equation (15) requires that $\beta_{q^{*}}<\psi$ and $\beta_{q^{\prime }}>\psi ,\;q^{\prime }\in \left\{1,2,\cdots ,Q\right\}/q^{*}$, where ψ is the threshold used to guarantee the maximum tolerable error in localizing the *q*-th SUE. If $\beta_{q^{*}}>\psi$, the *q*-th SUE fails to find the grid it is camping in and has to search the WFPD with its newly measured fingerprint again. It is worth mentioning that depending on the grid area and the error threshold ψ, WFP duplications [29] may be encountered by the SUEs. This situation usually results from the small grid area and a high ψ setting in practice. In this case, the WFP duplications would not give rise to wrong spectrum decisions because the grids that have the same WFP will have the same spectrum label as well.

### 4.3. Grid Oriented Spectrum Decision Making

In the previous subsection, the *q*-th SUE finds its location in the $q^{*}$-th grid via WFP matching in the WFPD. In order to determine which region the $q^{*}$-th grid belongs to, the distances between the $q^{*}$-th grid and the active PUTs need to be evaluated. However, calculating the distances demands geolocation information of the $q^{*}$-th grid and the active PUTs, whereas in practice $C_{p}^{\mathrm{PUT}}$ may be either readily available or completely unknown for the SUEs. It actually imposes two different situations to be dealt with by different approaches.

#### 4.3.1. PUT’s Geo-Location Information Aided Spectrum Decision Making

When the locations of the PUTs in the CCRN are readily known to the SUEs, it is set in the WFPD that PUT_Position_Flag=1. Once the location of the *q*-th SUE is determined as in the $q^{*}$-th grid, the flag $\mathrm{Region}\_\mathrm{Flag}\_q^{*}$ can be immediately determined, depending on its distance to the PUTs and the predefined region radius $D_{t}$ and $D_{p}$, as

(16) $$\begin{array}{c} \mathrm{Region}\_\mathrm{Flag}\_q^{*}=\left\{\begin{array}{ccc} 1 & \; & \;\;\;\;\;\;\sum_{p=1}^{P}∥C_{p}^{\mathrm{PUT}}-C_{q^{*}}^{\mathrm{SUE}}∥\cdot I\left({\hat{E}}_{p,h}=E_{p}\right)<{∥{\hat{P}}_{h}∥}_{0}\cdot D_{t},\\ 0 & \; & \;\;\;\;∥{\hat{P}}_{h}{∥}_{0}\cdot D_{t}\leq \sum_{p=1}^{P}∥C_{p}^{\mathrm{PUT}}\;-\;C_{q^{*}}^{\mathrm{SUE}}∥\cdot I\left({\hat{E}}_{p,h}=E_{p}\right)\;\leq \;{∥{\hat{P}}_{h}∥}_{0}\cdot D_{p},\\ -1 & \; & \;\;\;\;\;\;\sum_{p=1}^{P}∥C_{p}^{\mathrm{PUT}}-C_{q^{*}}^{\mathrm{SUE}}∥\cdot I\left({\hat{E}}_{p,h}=E_{p}\right)>{∥{\hat{P}}_{h}∥}_{0}\cdot D_{p}, \end{array}\right. \end{array}$$

where ${\hat{P}}_{h}$ is the power vector corresponding to the PUTs’ current operating state classified by the machine learning algorithms as ${\hat{C}}_{q^{*}}=h$, ${\hat{E}}_{p,h}$ is the power level accredited for the *p*-th PUT according to the *h*-th state of the *P* PUTs, and ${∥\;\cdot \;∥}_{0}$ is the $l_{0}$-norm. As for the label $\mathrm{Region}\_\mathrm{Flag}\_q^{*}$, $\mathrm{Region}\_\mathrm{Flag}\_q^{*}=1$ means that the SUE in the $q^{*}$-th grid is strictly prohibited from accessing the LFB $F_{2}$, $\mathrm{Region}\_\mathrm{Flag}\_q^{*}=0$ implies that the $q^{*}$-th grid is inside in the grey area and the SUEs inside it might cause interference to the potential PU receivers in its vicinity when they transmit over $F_{2}$, and $\mathrm{Region}\_\mathrm{Flag}\_q^{*}=-1$ claims that the $q^{*}$-th grid is sufficiently far away from any active PUT in the CCRN and hence the SUEs in the $q^{*}$-th grid can freely gain access to the LFB without yielding any interference to the PU receivers in the proximity of themselves.

**Figure 3:**
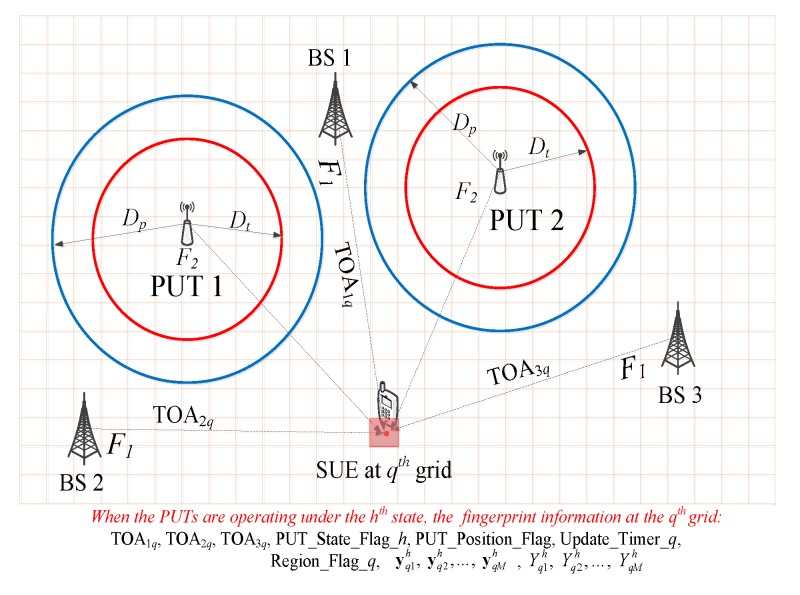
Grid-based sensing scenario and data collection.

In this way, the unavailable/available/uncertain status of the LFB $F_{2}$ can be determined as $\mathrm{PUT}\_\mathrm{Flag}\_q^{*}=1/-1/0$, respectively, depending on the region that the $q^{*}$-th grid belongs to. In the proposed sensing scheme, $\mathrm{PUT}\_\mathrm{Flag}\_q^{*}$ serves as the final sensing decision for the SUE located in the $q^{*}$-th grid in the CCRN. The $\mathrm{Update}\_\mathrm{Timer}\_q^{*}$ in Table 2 counts the time since the last update operation of $\mathrm{PUT}\_\mathrm{Flag}\_q^{*}$. When it reaches an upper limit φ, it automatically returns to zero and sets the $\mathrm{PUT}\_\mathrm{Flag}\_q^{*}$ as 0, meaning that the status of the PUT needs to be reconfirmed. If no data can be obtained for updating the $\mathrm{PUT}\_\mathrm{Flag}\_q^{*}$, it is better to keep it as 0 to prevent possible interference to the PU receivers. It is of particular importance to note that when $\mathrm{PUT}\_\mathrm{Position}\_\mathrm{Flag}\_q^{*}=0$, the $\mathrm{PUT}\_\mathrm{Flag}\_q^{*}$ needs to be set as 0, meaning that Equation (16) is not applicable due to the lack of $C_{p}^{\mathrm{PUT}}$. It is therefore necessary to determine whether the LFB $F_{2}$ is accessible by the *q*-th SUE, by means of either conventional sensing methods or MLSS algorithms. In other words, without knowledge of the PUTs’ locations, the SUEs are no longer able to decide the region that their locations belong to, but have to merely rely on the received signal energy to identify the status of the $F_{2}$. Figure 3 gives a depiction of the grid oriented spectrum sensing scenario, where the *q*-th SUE first performs TOA estimation based on the received reference signals from the three BSs in the CCRN, and it is then able to locate itself through WFP matching operation in the WFPD. Subsequently, the *q*-SUE can identify its spectrum label from the WFP it matches in the WFPD or through different grid oriented spectrum decision making methods described within this subsection.

#### 4.3.2. Machine Learning Aided Spectrum Decision Making

Intrinsically as classifiers or clustering algorithms, typical ML methods, e.g., SVM [34], K-means [35], and K-nearest neighbors (KNN) [36], can be employed for identifying the $\mathrm{PUT}\_\mathrm{Flag}\_q^{*}$ for the *q*-th SUE. In the sequel, we take the SVM classification algorithm as an example (Compared to the SVM algorithm adopted for spectrum decision making, the proposed scheme can be implemented with the K-means and KNN algorithms in a similar workflow. The difference between employing SVM and K-means lies in that the SVM algorithm requires a readily available label set in training whereas the K-means algorithm only demands the number of clusters in training, significantly relieving the implementation requirement.) and describe how the spectrum sensing decision is made for the SUEs in the *q*-th grid.

Similar to the conventional MLSS methods, we utilize the training data set and the corresponding label set as

(17) $$\begin{array}{cc} \Theta_{q,L} & =\left\{\theta_{q}^{\left(1\right)},\theta_{q}^{\left(2\right)},\cdots ,\theta_{q}^{\left(l\right)},\cdots ,\theta_{q}^{\left(L\right)}\right\},\\ C_{q,L} & =\left\{C_{q}^{\left(1\right)},C_{q}^{\left(2\right)},\cdots ,C_{q}^{\left(l\right)},\cdots ,C_{q}^{\left(L\right)}\right\}, \end{array}$$

where the training label set $C_{q,L}$ is different form the training label set $C_{q,L}$ in Equation (9) in that $C_{q}^{\left(l\right)}\in \left\{0,1,\cdots ,2^{P}\;-\;1\right\}$.

**Figure 4:**
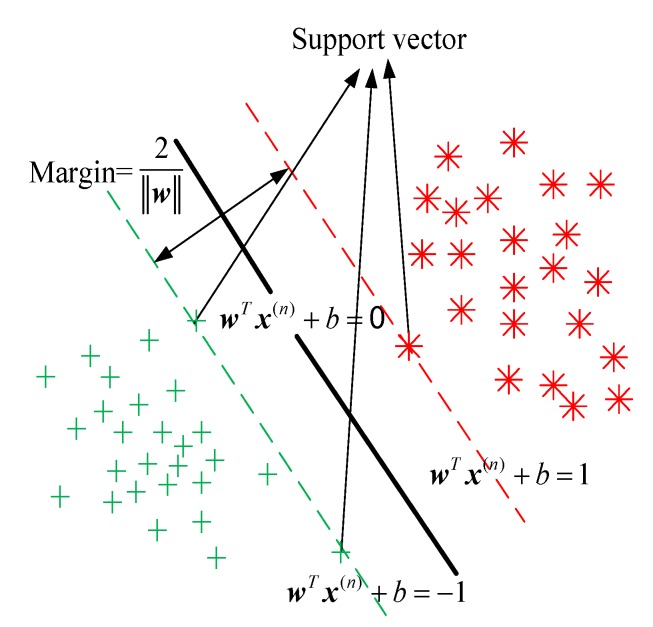
Support vector machine (SVM) model © 2019 IEEE [28].

The SVM originally provides a binary model in machine learning which strives to find a linearly separable hyperplane with the help of support vectors that lie closest to the decision surface by maximizing the margin of the classifier while minimizing the sum of classification errors [24], as shown in Figure 4, where $x^{\left(n\right)}$ is the *n*-th training sample, $x^{*(s)}$ is the *s*-th test sample, $y^{\left(n\right)}$ and $y^{*(s)}$ are, respectively, their corresponding labels, and the number of training samples and test samples is *N* and *S*, respectively.

The learning strategy of SVM is to maximize the margin shown in Figure 4 and its learning goal is to find a hyperplane in the multi-dimensional samples space. The hyperplane equation can be expressed as

(18) $$\omega^{T}x^{\left(n\right)}+b=0,$$

where ω is the weighting vector and *b* is the bias.

During training, the SVM should satisfy the following condition for all n=1,2,⋯,N as

(19) $$y^{\left(n\right)}=\left\{\begin{array}{cc} 1, & \mathrm{if}\;\omega^{T}x^{\left(n\right)}+b\geq 1,\\ -1, & \mathrm{if}\;\omega^{T}x^{\left(n\right)}+b\leq -1. \end{array}\right.$$

We need to minimize the vector norm of ω so as to maximize the margin, and hence the objective function is

(20) $$\begin{array}{c} \mathrm{Min}\;\frac{1}{2}{∥\omega ∥}^{2}\\ s.t.\;y^{\left(n\right)}\left(\omega^{T}x^{\left(n\right)}+b\right)\geq 1. \end{array}$$

where ω and *b* in the optimal hyperplane can be obtained by solving the objective function.

In practice, when a test sample $x^{*(s)}$ is fed into the SVM model, the SVM can determine which class it belongs to through the following rules

(21) $${\hat{y}}^{*(s)}=\left\{\begin{array}{cc} 1, & \mathrm{if}\;\omega^{T}x^{*(s)}+b\geq 1,\\ -1, & \mathrm{if}\;\omega^{T}x^{*(s)}+b<-1, \end{array}\right.$$

where ${\hat{y}}^{*(s)}$ is the predicted label of the *s*-th binary test sample.

However, practically the samples are not always linearly separable. For this case, the hyperplane satisfying such conditions does not exist at all. Then, we need to find a fixed nonlinear mapping function ϕ(.) to map the non-linear samples into a new feature space and use a linear SVM in the feature space [34]. Hence, the nonlinear SVM should satisfy the following condition for all n=1,2,⋯,N as

(22) $$y^{\left(n\right)}=\left\{\begin{array}{cc} 1, & \mathrm{if}\;\omega^{T}\phi \left(x^{\left(n\right)}\right)+b\geq 1,\\ -1, & \mathrm{if}\;\omega^{T}\phi \left(x^{\left(n\right)}\right)+b\leq -1. \end{array}\right.$$

The decision rule for nonlinearly SVM is given as

(23) $${\hat{y}}^{*(s)}=\left\{\begin{array}{cc} 1, & \mathrm{if}\;\omega^{T}\phi \left(x^{*(s)}\right)+b\geq 1,\\ -1, & \mathrm{if}\;\omega^{T}\phi \left(x^{*(s)}\right)+b<-1. \end{array}\right.$$

While the training energy vectors have been mapped into a higher dimensional feature space, practically we cannot achieve a perfect linearly separable hyperplane that satisfies the condition in Equation (20) for each $x^{\left(n\right)}$. Therefore, we rewrite the optimization problem as a convex optimization problem as follows

(24) $$\begin{array}{c} \mathrm{Min}\;\frac{1}{2}{∥\omega ∥}^{2}+\Lambda \sum_{n=1}^{N}\xi^{\left(n\right)}\\ s.t.\;y^{\left(n\right)}\left(\omega^{T}x^{\left(n\right)}+b\right)\geq 1-\xi^{\left(n\right)},n=1,2,\cdots ,N\\ \;\;\;\;\;\;\;\;\;\xi^{\left(n\right)}\geq 0,n=1,2,\cdots ,N, \end{array}$$

where Λ is a soft margin constant, for which a larger Λ means the assignment of a higher penalty to errors, and $\xi^{\left(n\right)}$ is a slack variable. As it is well known that the Radial basis function (rbf) kernel is a popular kernel function used in various kernelized learning algorithms to map a feature space to a higher dimension, it is adopted for the SVM classifier in this paper.

In order to obtain the final spectrum sensing decision for the SUEs located in the $q^{*}$-th grid, $\mathrm{PUT}\_\mathrm{Flag}\_q^{*}$, there are two types of SVM classifiers that need to be utilized in two consecutive stages. The first type SVM classifier, denoted as T1-SVM, is first evoked to classify the current operating state of the PUTs as ${\hat{C}}_{q^{*}}\in \left\{0,1,\cdots ,2^{P}-1\right\}$ and the second type SVM classifier, denoted as T2-SVM, is subsequently triggered to identify the availability of the LFB $F_{2}$ as $\mathrm{PUT}\_\mathrm{Flag}\_q^{*}\in \left\{1,0,-1\right\}$. For the training of the T1-SVM classifiers, it is assumed that there are enough number of SUEs pre-traversing all the grids in the CCRN and experiencing all the $2^{P}$ operating states of the *P* PUTs over a relatively long time, for example a duration of hundreds or even thousands of spectrum sensing periods $T_{s}$. In this way, $\Theta_{q,L}$ and $C_{q,L}^{\left(1\right)}$ are collected with a sufficiently large *L* and used as the training data set and label set for the T1-SVM classifier, respectively, with $C_{q,L}^{\left(1\right)}\in \left\{0,1,\cdots ,2^{P}-1\right\}$. For the T2-SVM classifier training, the classification models are trained under different operating states of the PUTs. Given the label $C_{q,L}^{\left(1\right)}=h$ from the T1-SVM, the training data set ${\overset{˘}{\Theta }}_{q,h}=\left\{{\overset{˘}{\theta }}_{q}^{\left(1\right)},{\overset{˘}{\theta }}_{q}^{\left(2\right)},\cdots ,{\overset{˘}{\theta }}_{q}^{\left(L_{h}\right)}\right\}\subset \Theta_{q,L}$ is taken from $\Theta_{q,L}$, with $C_{q,L}^{\left(1\right)}$ identifying the PUTs’ operating state that the data set ${\overset{˘}{\Theta }}_{q,h}$ is obtained under. The label set for T2-SVM training is $C_{q,h}^{\left(2\right)}$ with $C_{q,h}^{\left(2\right)}\in \left\{1,0,-1\right\}$. Specifically, for the training process, we have $\sum_{h=0}^{2^{P}-1}L_{h}=L$ and ${⋃}_{h=0}^{2^{P}-1}{\overset{˘}{\Theta }}_{q,h}=\Theta_{q,L}$. After training, the current operating state of the PUTs can be predicted by the T1-SVM classifier as a label ${\hat{C}}_{q}^{\left(1\right)}=h$, indicating that the current state is the *h*-th state. Subsequently, under the ${\hat{C}}_{q}^{\left(1\right)}$-th PUTs’ state, the T2-SVM operates to determine the final spectrum sensing decision $\mathrm{PUT}\_\mathrm{Flag}\_q^{*}$ as ${\hat{C}}_{q}^{\left(2\right)}$.

Unlike the binary SVM classifier presented in Equation (23), T1-SVM and T2-SVM are required to work as non-binary classifiers and hence they need to be modified for the purpose of being able to make multiple classifications. We adopt a one-versus-all (OVA) [37,38] scheme to fulfill the multi-classification task. Without loss of generality, assuming that a four-class problem for the T1-SVM classification model is to be solved, i.e., P=2, we need a series of four SVM classifiers, denoted as $c_{1},c_{2},c_{3},$ and $c_{4}$, respectively. During the learning phase, the *i*-th binary classifier $c_{i}$ outputs a label ‘+1’ when it is determined that the training data belongs to the positive class, whereas it produces a label ‘-1’ when the training data is classified as being in the negative class. With four binary SVMs operating in the OVA scheme, the task of multi-classification can be completed. As shown in Table 3, the binary SVM classifier $c_{1}$ in the first row is trained by assigning the positive label to it while the remaining $c_{2},c_{3},$ and $c_{4}$ binary SVM classifiers are assigned the negative label. The classification label $l_{1}$ is actually a combination of the individual labels of the four classifiers in the first row and the label $l_{1}$ is represented by the label set of the four SVM classifiers as one final label. Similarly, for the second classifier $c_{2}$ in row 2 of Table 3, the positive label is assigned to it and the negative label is assigned to all the other classifiers. In general, for the $c_{i}$ classifier in the *i*-th row of Table 3, we assign the positive label to it and the negative label to the remaining classifiers in the $l_{i}$ row. In this way, the multiple classification task to obtain ${\hat{C}}_{q}^{\left(1\right)}$ and ${\hat{C}}_{q}^{\left(2\right)}$ can be fulfilled with different number of different binary SVM classifiers, respectively.

Due to the limited spectrum observation of the *q*-th SUE, $\theta_{q}$, it is straightforward to exploit spatial diversity to ameliorate the spectrum sensing accuracy through SUE cooperation. Specifically, we assume the $q^{\prime }$-th SUE $\left(q^{\prime }\in Q_{q}\right)$ stays in one of the $|Q_{q}|$ grids surrounding the *q*-th grid and they may have the same true $\mathrm{PU}\_\mathrm{Flag}\_q^{\prime },q^{\prime }\in Q_{q}$ with that of the *q*-th grid. Accordingly, the spectrum sensing scheme only depending on $\theta_{q}$ is defined in this paper as a single-SUE-based spectrum prediction scheme and the one depending on $\theta_{q}$ and the spectrum observations $\theta_{q^{\prime }},q^{\prime }\in Q_{q}$ from the SUEs in $Q_{q}$ as a joint-SUE spectrum prediction scheme.

For the single-SUE spectrum prediction, the spectrum sensing decision for the SUE located in the *q*-th grid is predicted as PU_Flag_q. For the joint-SUE spectrum prediction, a sum of the labels from the *q*-th grid and its neighboring grids in $Q_{q}$ is firstly obtained as

(25) $${\bar{\Lambda }}_{q}=\mathrm{PU}\_\mathrm{Flag}\_q+\sum_{t\in Q_{q}}\mathrm{PU}\_\mathrm{Flag}\_t,$$

and the final decision PU_Flag_q is made as

(26) $$\mathrm{PU}\_\mathrm{Flag}\_q\;=\left\{\begin{array}{ccc} -1 & \; & {\bar{\Lambda }}_{q}\leq {\bar{\eta }}_{1}\\ 0 & \; & {\bar{\eta }}_{1}<{\bar{\Lambda }}_{q}<{\bar{\eta }}_{2}\\ 1 & \; & {\bar{\Lambda }}_{q}\geq {\bar{\eta }}_{2} \end{array}\right.$$

where ${\bar{\eta }}_{1}$ and ${\bar{\eta }}_{2}$ are the lower and upper integer threshold designed for the joint-SUE prediction, respectively.

The proposed geolocation information aided spectrum sensing scheme is described in detail in Algorithm 1.
**Algorithm 1** Geo-location information aided spectrum sensing.**Input:**$\Theta_{q,L}$, $C_{q,L}$, $T_{qk}$, WFPD. % for the *q*-th SUE, q=1,2,⋯,Q **Output:**PUT_Flag_q. 1:train the *q*-th T1-SVM classifier via $\left\{\Theta_{q,L},C_{q,L}^{\left(1\right)}\right\}$ 2:train the *q*-th T2-SVM classifier via ${\left\{{\overset{˘}{\Theta }}_{q,h},C_{q,h}^{\left(2\right)}\right\}}_{h=0}^{2^{P}-1}$ 3:the *q*-th SUE estimates *K* TOAs (fingerprint) from the *K* BSs over $F_{1}$ 4:**if**$\beta_{q^{*}}<\psi$ and it satisfies (15) **then** 5:    the estimated fingerprint best matches the $q^{*}$-th WFP in the WFPD 6:    the *q*-th SUE is located in the $q^{*}$-th grid 7:**else** return to step 2 8:**end if** 9:the *q*-th SUE reads the labels in the $q^{*}$-th WFP and obtain10:   $\left\{\mathrm{PUT}\_\mathrm{Position}\_\mathrm{Flag}\_q^{*},\mathrm{Update}\_\mathrm{Timer}\_q^{*},\mathrm{PUT}\_\mathrm{Flag}\_q^{*}\right\}$11:**if**$\mathrm{PUT}\_\mathrm{Position}\_\mathrm{Flag}\_q^{*}=1$**then**12:    **if** $\mathrm{Update}\_\mathrm{Timer}\_q^{*}<\varphi$ **then**13:        **if** $\mathrm{PUT}\_\mathrm{Flag}\_q^{*}\neq 0$ **then**14:           output $\mathrm{PUT}\_\mathrm{Flag}\_q=\mathrm{PUT}\_\mathrm{Flag}\_q^{*}$15:           Exit to step 3316:        **end if**17:    **end if**18:**end if**19:**if**$\;\mathrm{Update}\_\mathrm{Timer}\_q^{*}=\varphi$ or $\mathrm{PUT}\_\mathrm{Flag}\_q^{*}=0$ **then** the *q*-th SUE20:    % single-SUE prediction21:    obtains the sensing observation $\theta_{q}$ over the LFB $F_{2}$22:    predicts the state of the PUTs as ${\hat{C}}_{q^{*}}^{\left(1\right)}$ via $\theta_{q}$ and the trained T1-SVM classifier23:    predicts $\mathrm{PUT}\_\mathrm{Flag}\_q^{*}$ as ${\hat{C}}_{q^{*}}^{\left(2\right)}$ via $\theta_{q},{\hat{C}}_{q^{*}}^{\left(1\right)}$ and the trained T2-SVM classifier24:    **if** Joint-SUE prediction is required **then**25:        reads received signals of the $|Q_{q}|$ grids that neighbor the $q^{*}$-th grid26:        predicts $\mathrm{PUT}\_\mathrm{Flags}\_q^{\prime },q^{\prime }\in \left\{Q_{q}\right\}$ independently, via single-SUE prediction27:        obtains $\mathrm{PUT}\_\mathrm{Flag}\_q^{*}$ by HDF voting based on $\mathrm{PUT}\_\mathrm{Flags}\_q^{\prime },q^{\prime }\in \left\{Q_{q}\cup \left\{q^{*}\right\}\right\}$28:    **end if**29:    update $\mathrm{PUT}\_\mathrm{Flag}\_q^{*}$ in the $q^{*}$-th WFP30:    clear and restart $\mathrm{Updata}\_\mathrm{Timer}\_q^{*}$ in the $q^{*}$-th WFP31:**end if**32:output $\mathrm{PUT}\_\mathrm{Flag}\_q=\mathrm{PUT}\_\mathrm{Flag}\_q^{*}$33:**if** A new sensing request occurs **then** return to step 235:Exit

Similar to Equation (10), the spectrum sensing accuracy of the proposed scheme is

(27) $$\begin{array}{cc} P_{A} & =\sum_{h=0}^{2^{P}-1}E_{q}\left[Pr\left(H_{h}\right)Pr\left({\hat{C}}_{q}^{\left(1\right)}=C_{q}^{\left(1\right)}|H_{h}\right)Pr\left({\hat{C}}_{q}^{\left(2\right)}=C_{q}^{\left(2\right)}|{\hat{C}}_{q}^{\left(1\right)}=C_{q}^{\left(1\right)},H_{h}\right)\right]\\ \; & \approx \frac{1}{Q}\sum_{q=1}^{Q}\sum_{h=0}^{2^{P}-1}Pr\left(H_{h}\right)I\left({\hat{C}}_{q}^{\left(2\right)}=C_{q}^{\left(2\right)},{\hat{C}}_{q}^{\left(1\right)}=C_{q}^{\left(1\right)}|H_{h}\right), \end{array}$$

where $H_{h}$ is the *h*-th hypothesis indicating that the PUTs operate under the *h*-th state.

In order to compare the average energy consumption of the *q*-th SUE in the conventional non-geolocation aided spectrum sensing scheme and the proposed geolocation-based spectrum sensing scheme, we adopt the following energy consumption calculation as

(28) $$\begin{array}{c} E_{q,\mathrm{avg}}=\;\;\lim_{N=(N_{1}+N_{2})\to \infty }\;\frac{1}{N_{1}\;+\;N_{2}}\left[\underset{E_{1}}{\underset{︸}{\left(N_{1}\;+\;N_{2}\right)E_{q,F_{1}}+\left(N_{1}\;+\;N_{2}\right)E_{\mathrm{WFP},F_{1}}}}\;+\;\underset{E_{2}}{\underset{︸}{v_{q,F_{2}}N_{2}\cdot \left(E_{\mathrm{scan},F_{2}}M\;+\;E_{\mathrm{report},F_{1}}\right)}}\right], \end{array}$$

where $E_{q,F_{1}}$ is the power consumption of the *q*-th SUE in routine operations such as synchronization and signal measurement over the $F_{1}$ frequency band, $E_{\mathrm{WFP},F_{1}}$ is the power consumed in WFP matching operations in the WFPD, $E_{\mathrm{scan},F_{2}}$ is the power consumed in capturing energy observations of spectrum samples over the LFB $F_{2}$, $E_{\mathrm{report},F_{1}}$ is the power consumption of reporting the spectrum observations to the *q*-th SUE’s serving BS for classification operations, $v_{q,F_{2}}\in \left\{0,1\right\}$ indicates whether the *q*-th SUE identifies the status of the LFB $F_{2}$ through the WFP matching procedure ($v_{q,F_{2}}=0$) or it is required to make a decision for the LFB $F_{2}$ spectrum ($v_{q,F_{2}}=1$) based on the spectrum observations, $N_{1}$ and $N_{2}$ are, respectively, the number of operations performed over a relatively long period of time, corresponding to the $E_{1}$ and $E_{2}$ power consumption. As it is easy to observe that when $v_{q,F_{2}}=0$, the power consumption of $E_{2}$ is saved because the *q*-th SUE is capable of discerning the availability of $F_{2}$ based on the WFPD only. With a well established WFPD, periodical spectrum observation acquisition from the LFB $F_{2}$, which is intrinsically demanded by the non-geolocation-based spectrum sensing scheme, is thus fully circumvented and the power consumption is significantly reduced because instead of operating on dual frequency bands $F_{1}$ and $F_{2}$ simultaneously, the *q*-th SUE only operates on the single frequency band $F_{1}$.

## 5. Simulation and Analysis

In this section, the performance of the proposed geolocation information aided spectrum sensing scheme is evaluated via Matlab 2016b and compared with the conventional sensing algorithms. The conventional scheme refers to the energy detection-based sensing scheme [6,7,15], including the AND and OR criteria-based hard decision fusion schemes. These schemes are claimed as conventional because they do not rely on the WFP matching mechanism but keep periodically scanning the licensed spectrum. We consider a 6 km × 6 km CCRN area consisting of 5625 grids, with the size for each grid as 80 m × 80 m. In simulations, the main parameters are given in Table 4, where their values are chosen under practical concern. For example, the minimum bandwidth of $F_{2}$ is set as 5 MHz, which is a good choice for the 4 G LTE technology-based SUEs. The time-frequency product Wτ is 500, which is sufficiently large for obtaining the energy sample.

**Figure 5:**
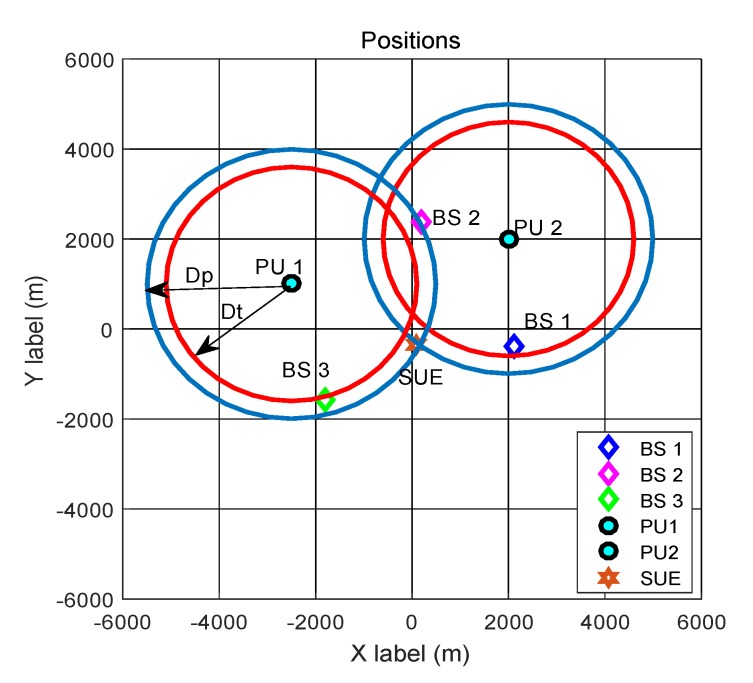
Spectrum sensing scenario in the cellular cognitive radio network (CCRN).

**Figure 6:**
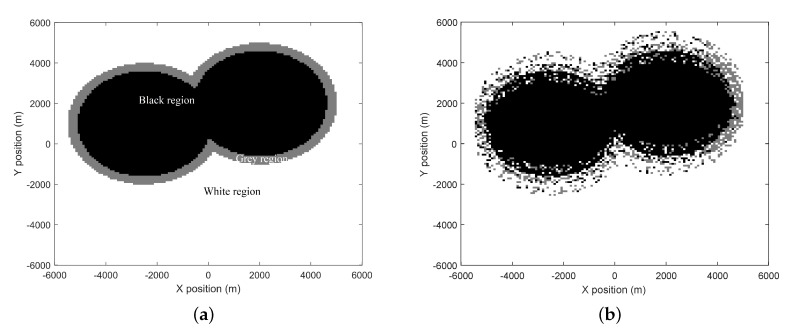
Region division when there are two active PUTs in the CCRN. (**a**) Regions ideally identified when there are two active PUTs in the CCRN. (**b**) Regions predicted when there are two active PUTs in the CCRN ($E_{P}$ = 5 W).

Figure 5 depicts the spectrum sensing scenario, where there are three BSs and two PUTs in the CCRN. When the two PUTs are both active, Figure 6a presents the ideally identified three regions for accessing the LFB $F_{2}$ and the regions predicted by the proposed algorithm are shown in Figure 6b. It is worth noting that Figure 6 is drawn from the grid oriented spectrum decisions, where the *q*-th grid in the figure is displayed as white, black, or grey according to PUT_flag_q∈{-1,1,0},q∈{1,2,⋯,Q}.

**Figure 7:**
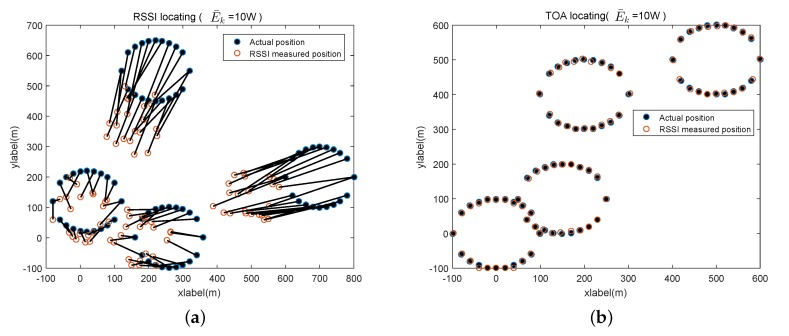
Accuracy comparison of TOA and RSS-based SUE positioning. (**a**) Accuracy of received signal strength (RSS)-based secondary user equipment (SUE) positioning. (**b**) Accuracy of time of arrival (TOA)-based SUE positioning.

For the WFPD aided SUE localization, we compare the positioning accuracy performance of the RSS and TOA schemes, as shown in Figure 7. It is easy to see that the differences between the actual positions and the estimated positions of the SUEs are apparent for the RSS scheme, whereas the differences are trivial for the TOA estimate-based scheme. It means that the accuracy of TOA-based positioning is much better than that of the RSS-based scheme, because the latter one is inherently more sensitive to the strength variations of the received reference signal over $F_{1}$.

**Figure 8:**
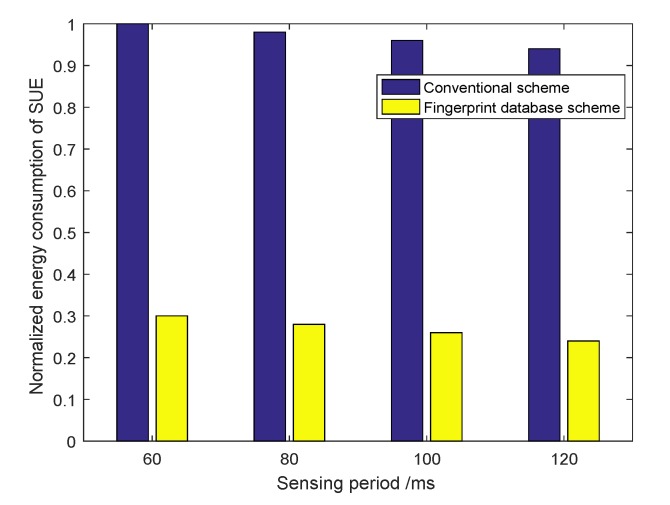
Power consumption of conventional sensing scheme and the wireless fingerprint database (WFPD) aided scheme with different sensing period $T_{s}$.

For the power efficiency, the conventional spectrum sensing scheme and the proposed WFP-based spectrum sensing scheme are compared in Figure 8. It is assumed that both $E_{\mathrm{scan},F_{2}}$ and $E_{\mathrm{WFP},F_{1}}$ are approximately equal to $E_{q,F_{1}}$, and $E_{\mathrm{report},F_{1}}$ is only a portion, 30%, of $E_{\mathrm{WFP},F_{1}}$. When the PUTs’ locations are already known to the SUEs or the spectrum availability information stored in the WFPD is within the newly updated period, the SUEs are able to determine the spectrum availability by searching the WFPD only. In other words, it is not necessary for the the SUEs to operate in the dual-band mode, and, therefore, the power consumptions of $E_{\mathrm{scan},F_{2}}$ and $E_{\mathrm{report},F_{1}}$ are totally saved. It is shown that the proposed scheme saves much more energy than the conventional non-WFPD aided sensing scheme. In the conventional sensing scheme, it requires the SUEs to keep sensing the LFB $F_{2}$ periodically whenever there is a request to access it, whereas for the proposed scheme, the SUEs only need to sense the spectrum on condition that the $F_{2}$ status in the WFPD is outdated or there is a request to obtain the spectrum observations over $F_{2}$.

**Figure 9:**
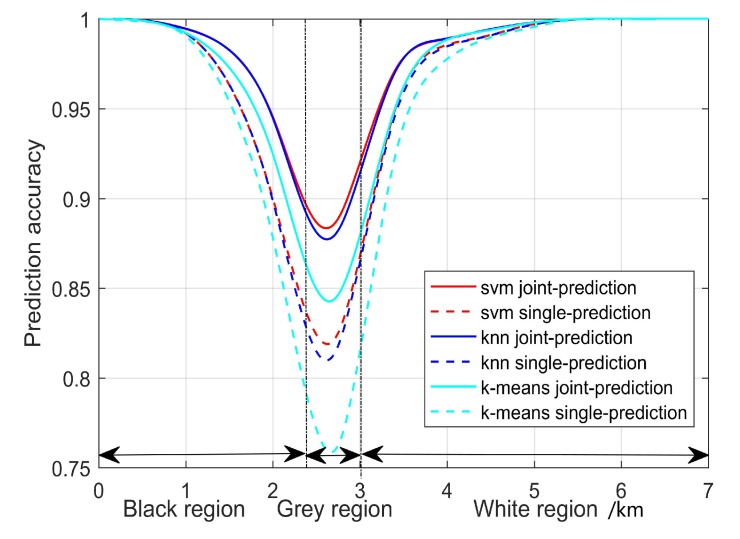
Prediction accuracy of different machine learning (ML) algorithms.

**Figure 10:**
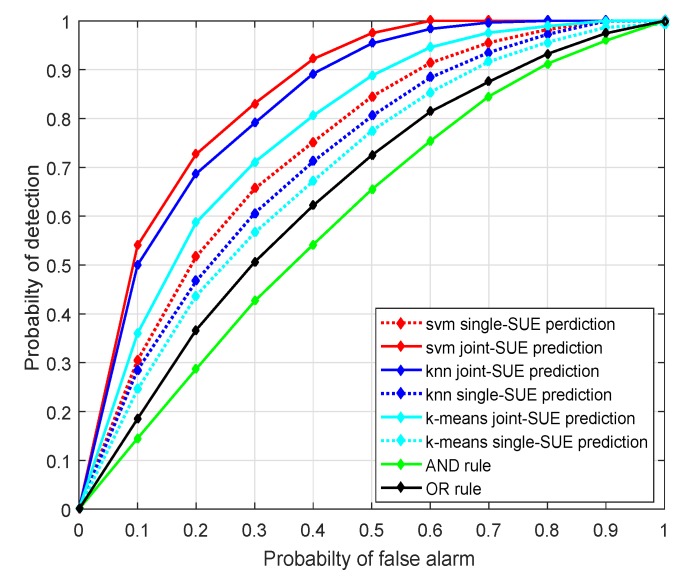
Receiver operating characteristics (ROC) of different sensing algorithms.

For the joint-SUE prediction-based sensing gain, we compare the spectrum prediction accuracies for the joint-SUE prediction with $|Q_{q}|=8$ and the single-SUE-based prediction, using SVM, KNN, and K-means algorithms. Figure 9 gives the prediction accuracy according to the distance between the SUE and one active PUT, where the PUT is located at the 0 km point and the grey region is about from 2∼3.5 km. It is shown that the prediction accuracy is not satisfactory within the grey region, because it suffers an ambiguity in discerning the data collected from the boundaries of the three regions. As shown, the prediction accuracy achieves 100% in the black and white regions and decreases when the SUE moves around the grey area. Due to the fact that the energy observations collected from the region borders are statistically indiscernible and it is hard for ML algorithms to classify them, the SUEs need to be conservative to gain spectrum opportunities in this region. We also compare KNN, SVM, K-means, and conventional sensing schemes in Figure 10, where the ROC curves for different schemes are depicted. The proposed SVM aided sensing scheme outperforms the other ML algorithms-based scheme and the conventional ED-based sensing schemes.

## 6. Conclusions

In this paper, a geolocation information-based spectrum sensing mechanism is proposed for the SUEs in the CCRN. By formulating the first task of sensing as identifying the positions of the SUEs through the WFP matching operation in the WFPD, we tackle the second task as ascertaining the grid oriented spectrum availability through either spectrum labels in the WFPD or ML algorithm aided spectrum observation classification. On the condition that the PUTs’ locations are readily known, the SUEs just need to check the LFB occupancy status in the WFPD or the distance between the SUE and the active PUTs, whereas when the PUTs’ locations are unknown, the SUEs have to gather data from their neighboring grids to obtain the final spectrum decision, with the help of MLSS algorithms. Simulation results verified that the TOA estimation-based WFP scheme is superior to the RSS-based scheme for the first task. As for the grid oriented spectrum decision making mechanism, the SVM algorithm is verified to achieve higher spectrum prediction accuracy than the KNN and K-means algorithms. Meanwhile, the proposed scheme exhibits the best performance in terms of detection probability, compared with the ED-based HDF methods. Since the problem of only two transmit power levels of the PUTs are investigated in this paper, the methods and analysis for the case of multiple power levels are to be addressed in future work. Due to its salient power-saving capability in sensing operations, the proposed geolocation information aided spectrum sensing scheme can be used as one practical candidate solution in the CCRN.

## Figures and Tables

**Table 1:** Region oriented spectrum availability.

Region Type	Region_Flag	PUT_Flag	Availability of $F_{2}$ for the SUEs
Black Region	1	1	strictly non-accessible
Grey region	0	0	uncertain
White region	-1	-1	freely accessible

**Table 2:** Data table of the time of arrival (TOA) wireless fingerprint database.

Data Type	WFP 1	WFP 2	⋯	WFP *q*	⋯	WFP *Q*
**Geo-Location**	$C_{1}^{\left(\mathrm{SUE}\right)}$	$C_{2}^{\left(\mathrm{SUE}\right)}$	⋯	$C_{q}^{\left(\mathrm{SUE}\right)}$	⋯	$C_{Q}^{\left(\mathrm{SUE}\right)}$
	${\mathrm{TOA}}_{11}$	${\mathrm{TOA}}_{12}$	⋯	${\mathrm{TOA}}_{1q}$	⋯	${\mathrm{TOA}}_{1Q}$
**WFP Feature**	${\mathrm{TOA}}_{21}$	${\mathrm{TOA}}_{22}$	⋯	${\mathrm{TOA}}_{2q}$	⋯	${\mathrm{TOA}}_{2Q}$
**(TOA)**	⋮	⋮	⋮	⋮	⋮	⋯
	${\mathrm{TOA}}_{K1}$	${\mathrm{TOA}}_{K2}$	⋯	${\mathrm{TOA}}_{Kq}$	⋯	${\mathrm{TOA}}_{KQ}$
**Spectrum**	Region_Flag_1	Region_Flag_2	⋯	Region_Flag_q	⋯	Region_Flag_Q
**Label**	PUT_Flag_1	PUT_Flag_2	⋯	PUT_Flag_q	⋯	PUT_Flag_Q
**Timer**	Update_Timer_1	Update_Timer_2	⋯	Update_Timer_q	⋯	Update_Timer_Q
**Most Recent**	$y_{11}^{h},y_{12}^{h},\cdots ,y_{1M}^{h}$	$y_{21}^{h},y_{22}^{h},\cdots ,y_{2M}^{h}$	⋯	$y_{q1}^{h},y_{q2}^{h},\cdots ,y_{qM}^{h}$	⋯	$y_{Q1}^{h},y_{Q2}^{h},\cdots ,y_{QM}^{h}$
**Observations**	$Y_{11}^{h},Y_{12}^{h},\cdots ,Y_{1M}^{h}$	$Y_{21}^{h},Y_{22}^{h},\cdots ,Y_{2M}^{h}$	⋯	$Y_{q1}^{h},Y_{q2}^{h},\cdots ,Y_{qM}^{h}$	⋯	$Y_{Q1}^{h},Y_{Q2}^{h},\cdots ,Y_{QM}^{h}$
**Public Labels**	PUT_State_Flag_*h*, $h\in \{0,1,\cdots ,2^{P}\;-\;1\}$; PUT_Position_Flag					

**Table 3:** Classification coding matrix of the one-versus-all (OVA) scheme.

	$c_{1}$	$c_{2}$	$c_{3}$	$c_{4}$
$l_{1}$	+1	-1	-1	-1
$l_{2}$	-1	+1	-1	-1
$l_{3}$	-1	-1	+1	-1
$l_{4}$	-1	-1	-1	+1

**Table 4:** Simulation parameters.

Parameter	Value
CCRN Area	6 km × 6 km
Grid Area	80 m × 80 m
Number of BSs, *K*	3
Number of Grids, *Q*	5625
Number of PUTs, *P*	2
Bandwidth of $F_{2}$, *W*	5 MHz
Sensing Interval, τ	100 μs
Sensing Period, $T_{s}$	100 ms
Maximum Time in Update_Timer_*q*, φ	$1000\;T_{s}$
Number of sensing segments, *M*	10
Pathloss component, α	4
Shadowing component, $\zeta_{p}$	2 dB
Fading component, $\nu_{p}$	5 dB
Length of data for T1-SVM training, *L*	22,500
Length of data for T2-SVM training, $L_{h}$	5625
